# Supplementary material for: ARMH2 is a cytosolic component of CatSper crucial for sperm function
Source: Nat Commun. 2025 Nov 21;16:10243. doi: 10.1038/s41467-025-65952-0 (PMC12638846; doi:10.1038/s41467-025-65952-0)
Supplement: Supplementary file 1 — Supplementary Information [file 41467_2025_65952_MOESM1_ESM.pdf]

Supplementary information for

ARMH2 is a cytosolic component of CatSper crucial for sperm function

Qingqing Zhao, Shiyi Lin, Hang Kang, Yanfei Ru, Qikui Xu, Zijing Yu, Xiaofang Huang, Carlo De Rito, Giulia Sassi, Shaojie Wang, Shuya Sun, Rui Sun, Honghan Cheng, Yi Zhu, Mingxi Liu, Yongdeng Zhang, Min Jiang, Riccardo Percudani, Jean-Ju Chung, Xuhui Zeng*, Zhen Yan*, Jianping Wu*

*Correspondence: wujianping@westlake.edu.cn (J.W.); yanzhen@westlake.edu.cn (Z.Y.); zengxuhui@ntu.edu.cn (X.Z.)

This file includes:

Supplementary Figs. 1-8

Supplementary Table 1

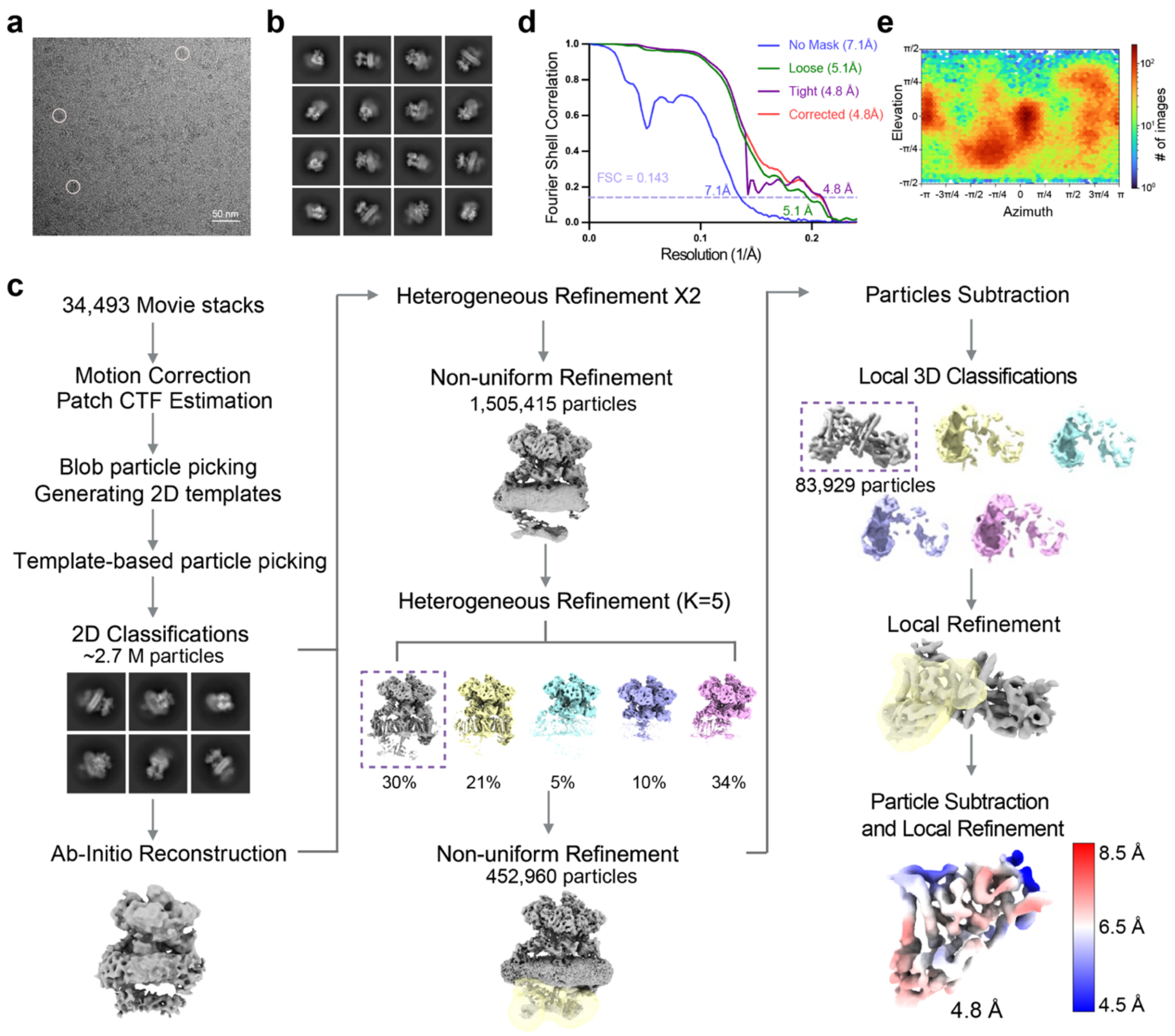

Supplementary Fig. 1. Cryo-EM analysis of the cytosolic region of CatSper. a, Representative cryo-EM micrograph selected from a dataset of 34,493 micrographs. A few particles are indicated by white circles. **b,** 2D class averages. Box size: 418 Å. **c,** Flowchart of EM data processing. See “Image processing” in the Methods section for details. **d-e,** Gold-standard Fourier shell correlation (FSC) curves (**d**) and angular distribution of particles (**e**) for the final cytosolic local reconstruction map, as shown in (**c**).

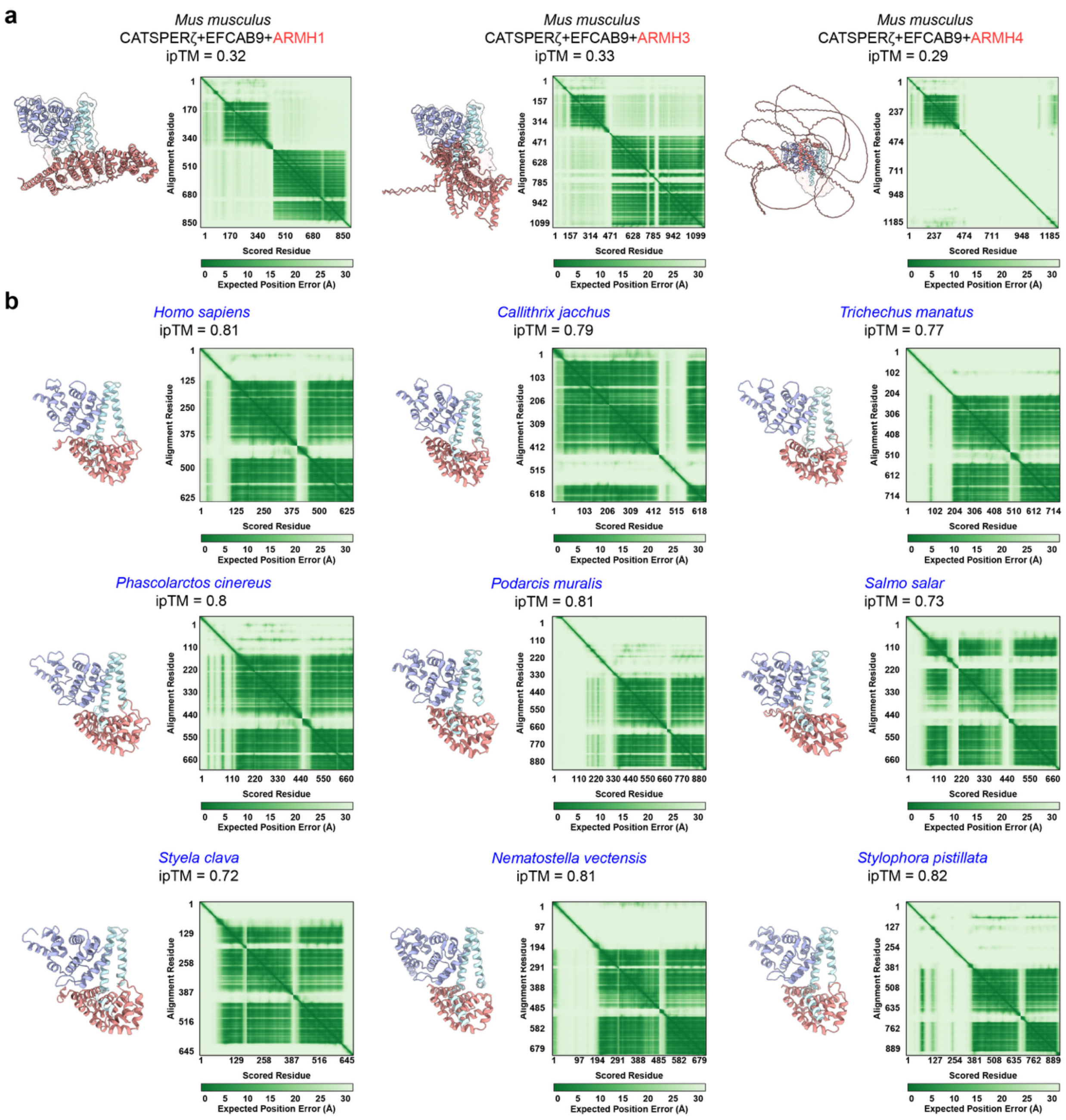

Supplementary Fig. 2. AlphaFold structure predictions for the cytosolic ternary subcomplex. a, AlphaFold 3 predicted structures of CATSPERζ (cyan) and EFCAB9 (slate) in complex with other ARMH family members (salmon), fitted within the context of cytosolic map 2. **b,** AlphaFold 3 predicted structures of the CATSPERζ-EFCAB9-ARMH2 subcomplex across different species. The ipTM score and the predicted aligned error are provided for each prediction.

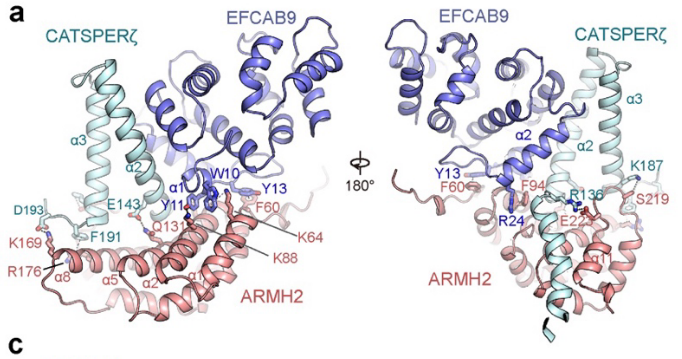

b

NCBI/Uniprot identifiers	ARMH2	EFCAB9	CATSPERζ
Mus musculus	A0A286YCZ6	Q9DAM2	NP_001034583.1
Homo sapiens	H3BNL8	A8MZ26	NP_001034585.1
Callithrix jacchus	F7I8V3	F7EGD7	F6TGP1
Trichechus manatus	A0A2Y9G340	A0A2Y9DAU7	A0A2Y9E0C7
Phascolarctos cinereus	A0A6P5KC84	A0A6P5KX65	A0A6P5IYI3
Podarcis muralis	A0A670ICB3	A0A670JI40	XP_028566054.1
Salmo salar	A0A8U0PDF2	A0A1S3RRB1	XP_014022385.2
Styela clava	XP_039267827	XP_039251933.1	XP_039258139.1
Nematostella vectensis	XP_032233047.2	A7RQT6	XP_032225778.1
Stylophora pistillata	A0A2B4RVC0	XP_022784613.1	XP_022790969.1

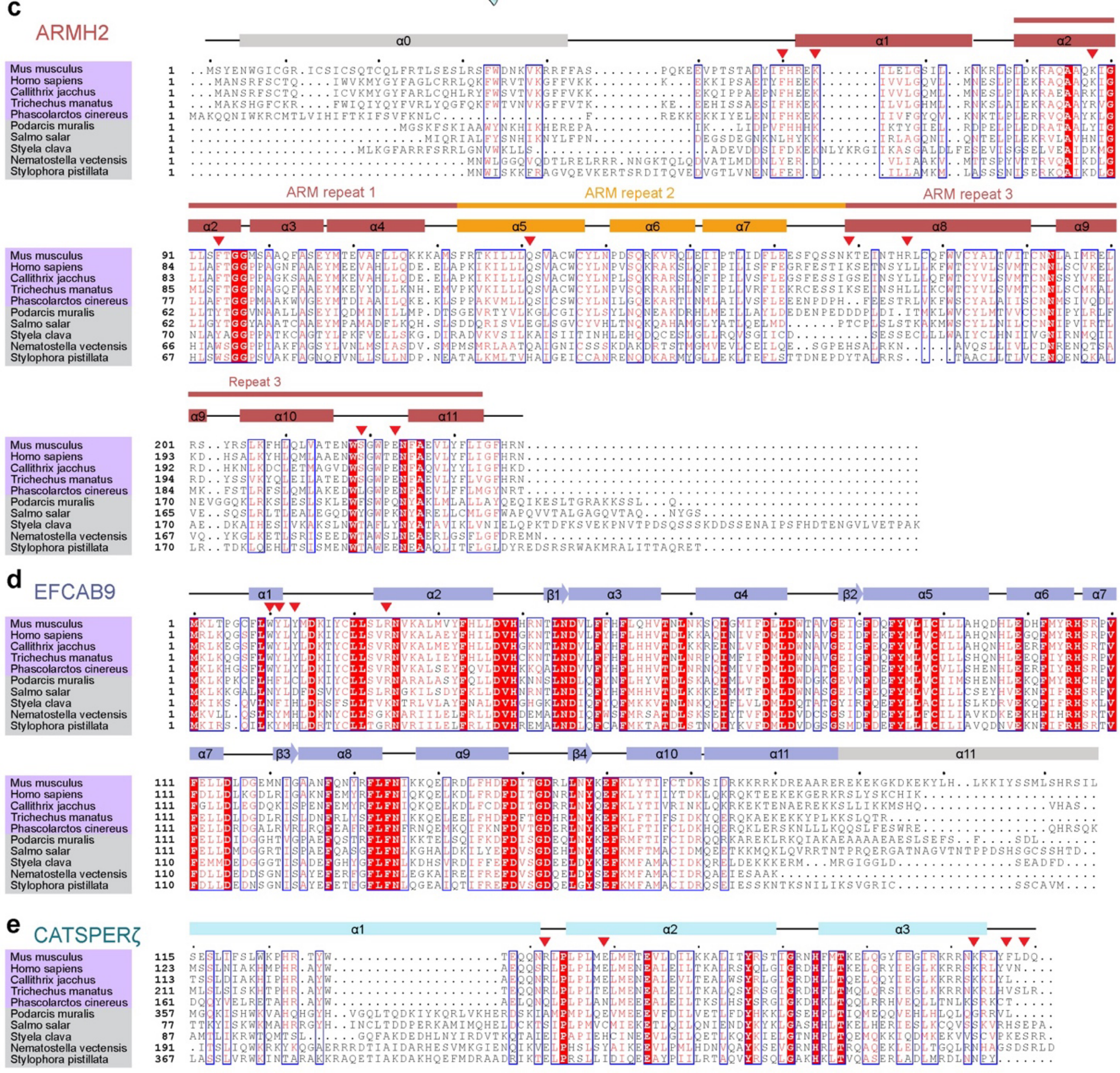

Supplementary Fig. 3. Interaction and multiple sequence alignments of the ARMH2-EFCAB9-CATSPERζ subcomplex. a, Potential specific interactions between ARMH2

(salmon) and EFCAB9 (slate) or CATSPERζ (cyan). Key residues involved in the potential interactions are shown in sticks and labelled. **b,** Summary of the NCBI or UniProt IDs of the protein sequences utilized for multiple sequence alignments. **c-e,** Multiple sequence alignment of ARMH2 (**c**), EFCAB9 (**d**), and CATSPERζ (**e**). Mammalian and non-mammalian species are shaded in purple and gray, respectively. The protein sequence alignment of ARMH2 was used to construct the domain HMM. Secondary structures, derived from the AlphaFold model of the mouse sequence, are represented above the alignment. Armadillo (ARM) repeats of ARMH2 determined by HHrepID are shown in different colors. The N-terminal of ARMH2 (α0) and a portion of the C-terminal of EFCAB9 (α11), predicted to form α helices, were not observed in the cryo-EM map and are colored gray. Residues that may be involved in specific interactions between ARMH2 and EFCAB9 or CATSPERζ are highlighted by red triangles.

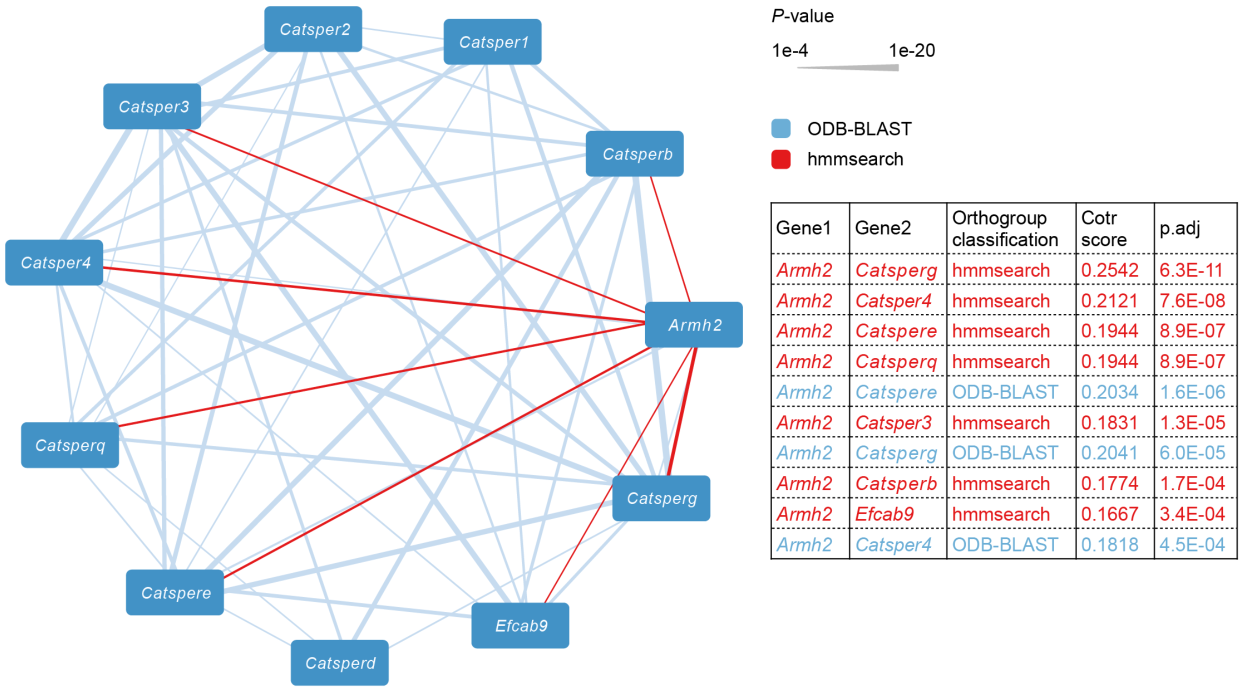

Gene1	Gene2	Orthogroup classification	Cotr score	p.adj
Armh2	*Catsperg*	hmmsearch	0.2542	6.3E-11
Armh2	*Catsper4*	hmmsearch	0.2121	7.6E-08
Armh2	*Catspere*	hmmsearch	0.1944	8.9E-07
Armh2	*Catsperq*	hmmsearch	0.1944	8.9E-07
Armh2	*Catspere*	ODB-BLAST	0.2034	1.6E-06
Armh2	*Catsper3*	hmmsearch	0.1831	1.3E-05
Armh2	*Catsperg*	ODB-BLAST	0.2041	6.0E-05
Armh2	*Catsperb*	hmmsearch	0.1774	1.7E-04
Armh2	*Efcab9*	hmmsearch	0.1667	3.4E-04
Armh2	*Catsper4*	ODB-BLAST	0.1818	4.5E-04

Supplementary Fig. 4. Network of co-evolutionary association among CatSper subunits. Edges represent significant Cotr scores between genes using orthogroup classification provided by OrthoDB V.11 (ODB-BLAST, light blue) or obtained through local HMM searches (hmmsearch, red). Edge thickness is inversely proportional to the *P* value as indicated in the table. *P* values were determined using Fisher exact test. p.adj indicates *P* values adjusted for multiple comparisons using the Holm correction.

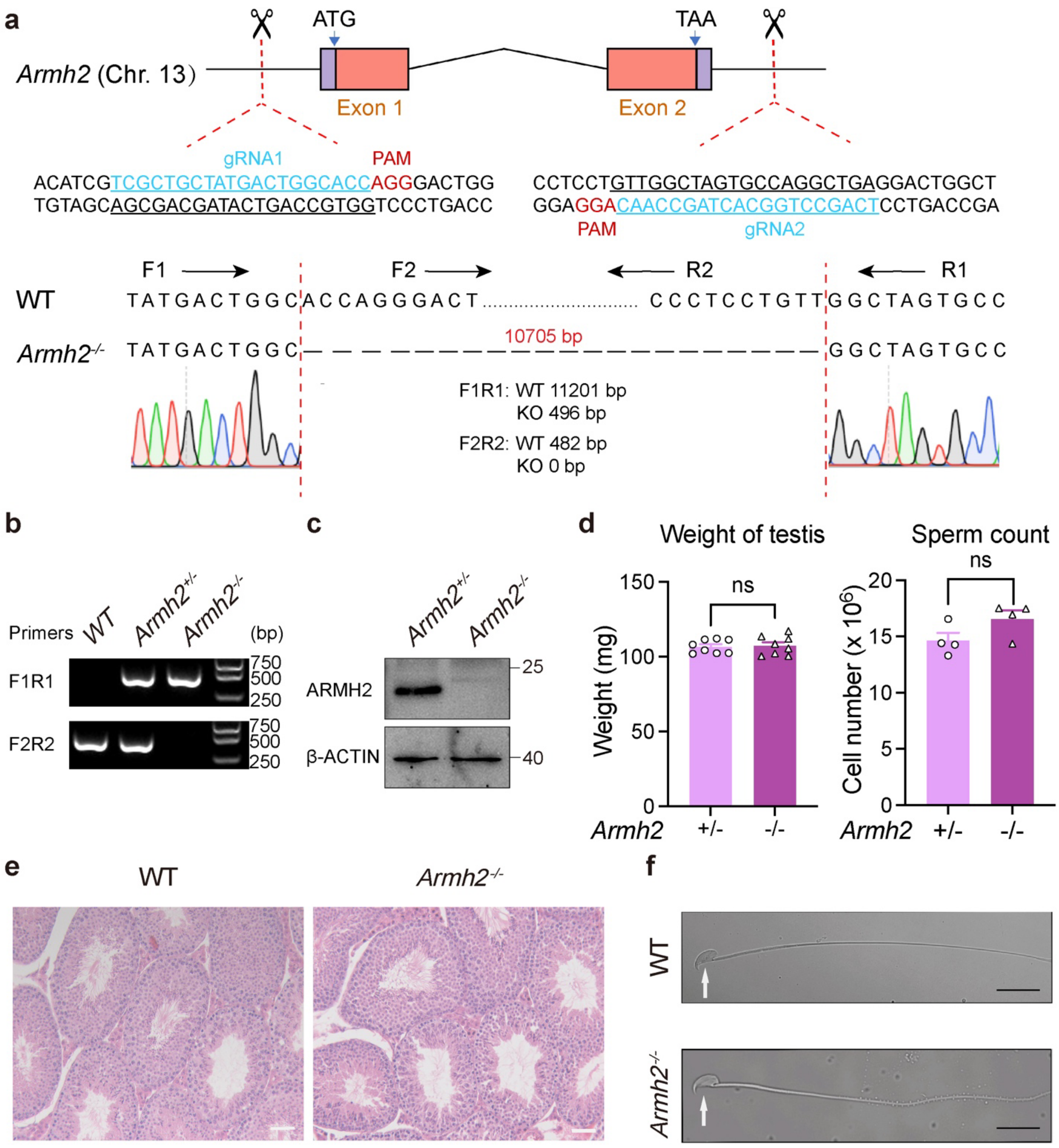

Supplementary Fig. 5. *Armh2* knockout does not influence gross phenotype. a, Generation of *Armh2* knockout mice using CRISPR/Cas9 technique. The knockout mouse exhibits a 10,705-bp deletion encompassing both exons of the *Armh2* gene, as confirmed by DNA sequencing. Red dash lines indicated two cutting sites, with the gRNA sequence and PAM regions highlighted in blue and red, respectively. **b,** Genotyping of the *Armh2* knockout mice by PRC using two primer pairs. **c,** Immunoblotting detection of ARMH2 protein in *Armh2*$^{+/-}$ and *Armh2*$^{-/-}$ sperm. **d,** The

measurement of testis weight and sperm count of *Armh2*$^{+/-}$ (light purple) and *Armh2*$^{-/-}$ (dark purple) male mice. n=4 for each group, where n represents the number of biologically independent replicates. Both testes from each male mouse were measured individually. Data are presented as mean ± SEM. Statistical significances were assessed using two-tailed unpaired Student's *t*-tests. The exact *P* values are as follows: weight of testis: 0.7993; sperm count: 0.1046. ns: no significant difference. Source data are provided in the Source Data file. **e,** Testis histology of WT and *Armh2*$^{-/-}$ male mice. Scale bar: 15 µm. **f,** Morphology of WT and *Armh2*$^{-/-}$ sperm imaged under bright field. Scale bar: 10 µm. The sperm heads are indicated by white arrows. The experiments in (**b**-**f**) were independently repeated for three times with similar results.

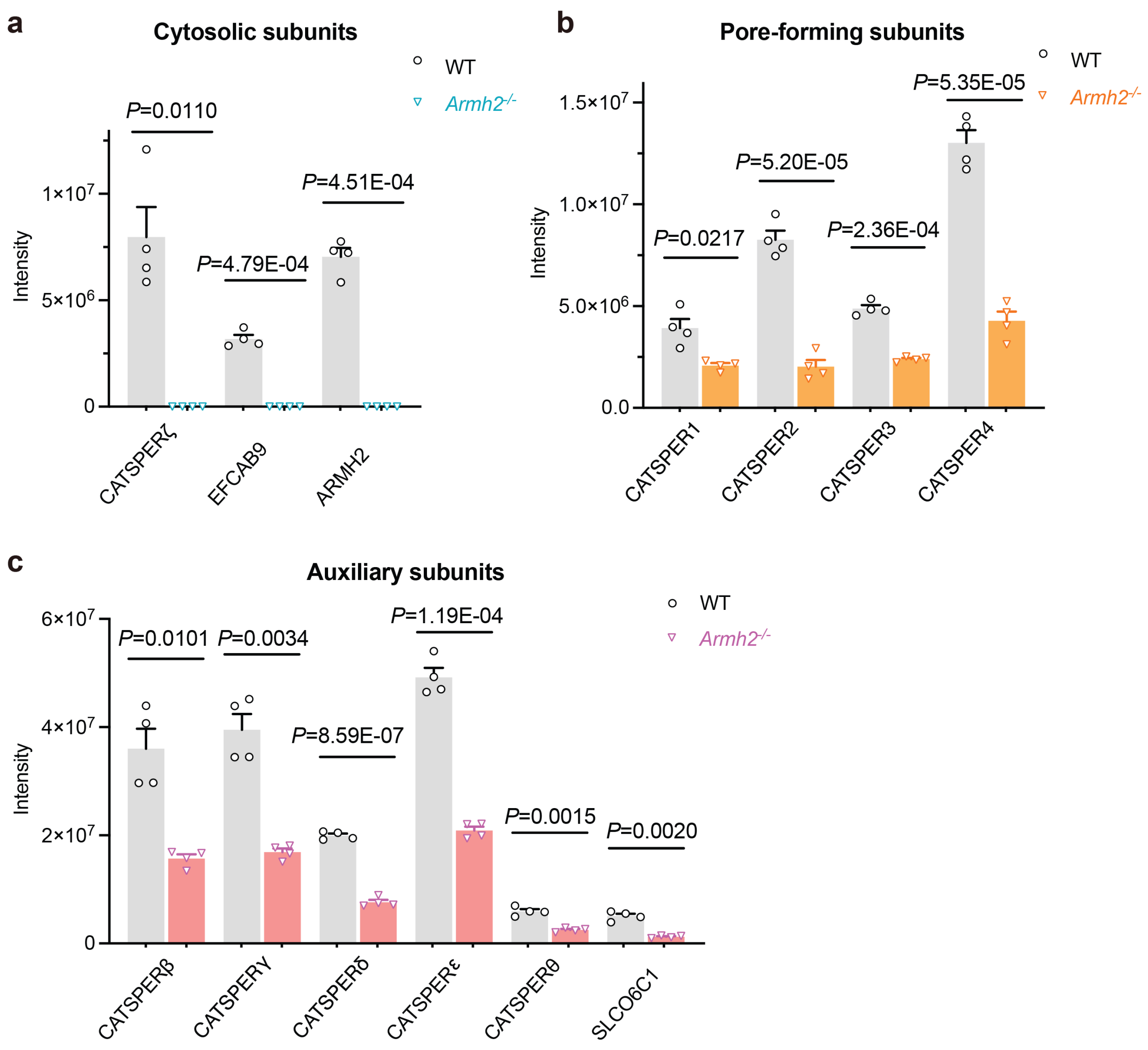

Supplementary Fig. 6. Lack of ARMH2 downregulates other CatSper subunits. Quantitative proteomic analysis of CatSper cytosolic subunits (**a**), pore-forming subunits (**b**) and transmembrane auxiliary subunits (**c**) between WT and *Armh2*$^{-/-}$ sperm. CATSPERη was not analyzed as it was not detected in both WT and *Armh2*$^{-/-}$ sperm samples, probably due to its small size. n=4 mice for each group. Data are presented as mean ± SEM. Statistical significances were assessed using two-tailed unpaired Student's *t*-tests. *P* values for each group are indicated in the figure. Source data are provided in the Source Data file.

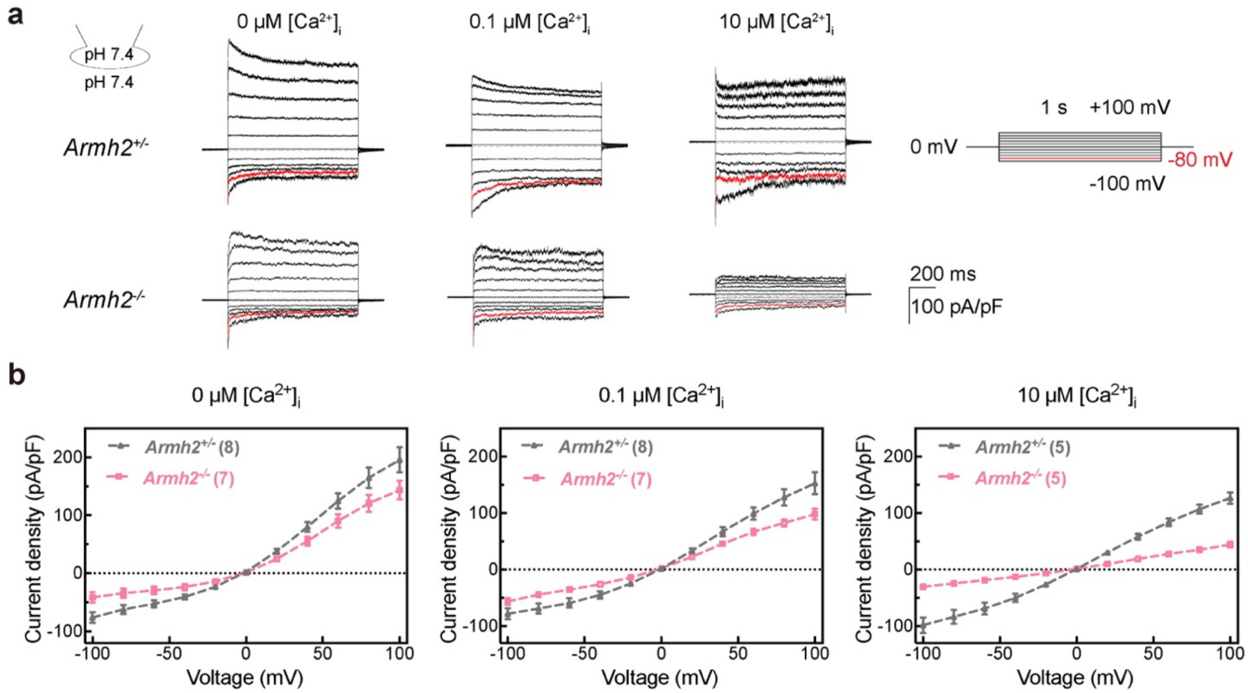

Supplementary Fig. 7. Step current recordings of $I_{CatSper}$ under different Ca^{2+} conditions. a, Representative step current traces of CatSper from *Armh2*$^{+/-}$ (upper) and *Armh2*$^{-/-}$ (lower) corpus sperm at an internal solution pH 7.4 with 0 µM, 0.1 µM, or 10 µM free Ca^{2+}. The step voltage stimulus ranged from −100 mV to +100 mV with a 20-mV increment for 1 s. Red highlighted traces were recorded at −80 mV. The results presented are representative of at least five independent replicate experiments. **b,** Current-voltage relationship (I-V curve) analysis of the step currents of CatSper at steady state, as recorded in (**a**). Biological replicate sperm numbers for each group were showed in brackets. Data are presented as mean ± SEM. Source data are provided in the Source Data file.

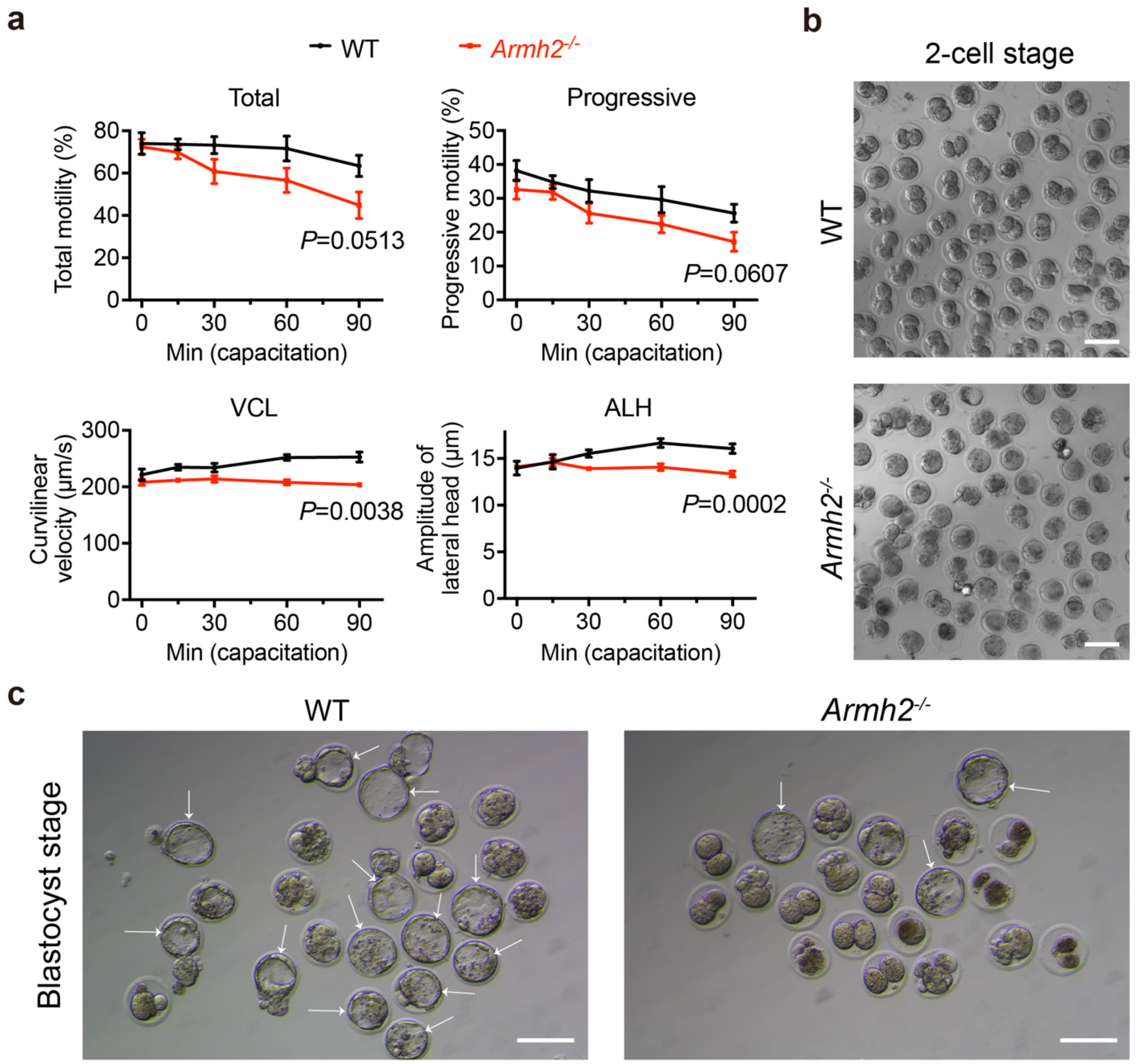

Supplementary Fig. 8. ARMH2 knockout affects sperm hyperactivated motility and male fertility. a, Motility parameters over time for WT (black) and *Armh2*$^{-/-}$ (red) sperm incubated in HTF medium, as measured by CASA. Data are presented as mean ± SEM, n=5 mice for each group. *P* values at 90 minutes capacitation time point for each group are indicated in the figure. Statistical significances were assessed using two-tailed unpaired Student's *t*-tests. Source data are provided in the Source Data file. **b-c,** Representative bright-field images of 2-cell embryos (day 2) (**b**) and blastocyst-stage embryos (day 5) (**c**) resulting from *in vitro* fertilization of WT oocytes with either WT or *Armh2*$^{-/-}$ sperm. Scale bars: 100 μm. White arrows indicate morphologically normal embryos at blastocyst stage. Representative images from three independent biological

replicate experiments are shown in (**b-c**). Multiple fields of view were acquired in each experiment, and a single representative image is presented.

Supplementary Table 1. Data processing and model statistics.

	ARMH2-EFCAB9-CatSperζ subcomplex (EMD-63452) (PDB:9LWO)
**Data collection and processing**	
Microscope	FEI Titan Krios
Magnification	81,000
Voltage (kV)	300
Detector	Gatan K3
Electron exposure (e^{-}/Å^{2})	50
Defocus range (μm)	-1.5 to -2.5
Pixel size (Å)	1.087
Symmetry imposed	C1
Final particle images (no.)	83,929
Map resolution (Å)	4.8
FSC threshold	0.143
**Refinement**	
Initial model used (PDB code)	AlphaFold 3 prediction
Map sharpening *B* factor (Å^{2})	-101.2
Model composition	
Non-hydrogen atoms	3,684
Protein residues	437
R.m.s. deviations	
Bond lengths (Å)	0.007
Bond angles (°)	1.920
Validation	
MolProbity score	1.92
Clashscore	9.05
Poor rotamers (%)	0.00
Ramachandran plot	
Favored (%)	93.27
Allowed (%)	6.50
Disallowed (%)	0.23
