# Supplementary material for: ARMH2 is a cytosolic component of CatSper crucial for sperm function
Source: Nat Commun. 2025 Nov 21;16:10243. doi: 10.1038/s41467-025-65952-0 (PMC12638846; doi:10.1038/s41467-025-65952-0)
Supplement: Supplementary file 2 — Description of Additional Supplementary Files [file 41467_2025_65952_MOESM2_ESM.pdf]

Description of Additional Supplementary Files

File Name: Supplementary Movie 1
Description: Super-resolution imaging of CatSper nanodomain. x-y projections are represented with the rotating angle around the x axis in a, and c, and y-z projections at cross-sections are played in b, and d, for WT and *Armh2*$^{-/-}$ sperm, respectively. Color code indicates z position.

File Name: Supplementary Movie 2
Description: Sperm waveform analysis. The spermatozoa were settled down to fibronectin-coated glass dishes. Sperm tail movement of uncapacitated (0 minute) and capacitated (90 minutes) cauda spermatozoa from *Armh2*$^{+/-}$ (a, b) and *Armh2*$^{-/-}$ (c, d) mice were recorded at 37°C at 200 fps. Each video is played at 100 fps (1/2 real speed).

File Name: Supplementary Data 1
Description: Proteomic analysis of WT and *Armh2*$^{-/-}$ sperm.
