# Supplementary material for: ARMH2 is a cytosolic component of CatSper crucial for sperm function
Source: Nat Commun. 2025 Nov 21;16:10243. doi: 10.1038/s41467-025-65952-0 (PMC12638846; doi:10.1038/s41467-025-65952-0)
Supplement: Supplementary file 6 — Reporting Summary [file 41467_2025_65952_MOESM6_ESM.pdf]

nature portfolio

Corresponding author(s): Jianping Wu, Zhen Yan, Xuhui Zeng

Last updated by author(s): Sep 25, 2025

Reporting Summary

Nature Portfolio wishes to improve the reproducibility of the work that we publish. This form provides structure for consistency and transparency in reporting. For further information on Nature Portfolio policies, see our Editorial Policies and the Editorial Policy Checklist.

Statistics

For all statistical analyses, confirm that the following items are present in the figure legend, table legend, main text, or Methods section.

n/a	Confirmed	
[ ]	[x]	The exact sample size (*n*) for each experimental group/condition, given as a discrete number and unit of measurement
[ ]	[x]	A statement on whether measurements were taken from distinct samples or whether the same sample was measured repeatedly
[ ]	[x]	The statistical test(s) used AND whether they are one- or two-sided<br>*Only common tests should be described solely by name; describe more complex techniques in the Methods section.*
[x]	[ ]	A description of all covariates tested
[x]	[ ]	A description of any assumptions or corrections, such as tests of normality and adjustment for multiple comparisons
[ ]	[x]	A full description of the statistical parameters including central tendency (e.g. means) or other basic estimates (e.g. regression coefficient) AND variation (e.g. standard deviation) or associated estimates of uncertainty (e.g. confidence intervals)
[ ]	[x]	For null hypothesis testing, the test statistic (e.g. *F*, *t*, *r*) with confidence intervals, effect sizes, degrees of freedom and *P* value noted<br>*Give P values as exact values whenever suitable.*
[x]	[ ]	For Bayesian analysis, information on the choice of priors and Markov chain Monte Carlo settings
[x]	[ ]	For hierarchical and complex designs, identification of the appropriate level for tests and full reporting of outcomes
[x]	[ ]	Estimates of effect sizes (e.g. Cohen's *d*, Pearson's *r*), indicating how they were calculated

Our web collection on statistics for biologists contains articles on many of the points above.

Software and code

Policy information about availability of computer code

Data collection	EPU2.12.0
Data analysis	OriginPro 2021b 9.8.5.204, MotionCor2, CryoSPARC 4.4.1, UCSF Chimera 1.17.3, UCSF ChimeraX 1.6.1, Coot 0.9.8.8, AlphaFold3, PHENIX 1.19.2, PyMOL 2.5.0, pFind3, pLink 2, FIJI, Zen 2.3 lite, Photoshop 24.3.0, PatchMaster, Igor Pro, Prism, Illustrator CS6, Biorender, Endnote X9, Cotr, Hmmer 3.3.2, Cytoscape 3.10.3, ggtree 3.1.0

For manuscripts utilizing custom algorithms or software that are central to the research but not yet described in published literature, software must be made available to editors and reviewers. We strongly encourage code deposition in a community repository (e.g. GitHub). See the Nature Portfolio guidelines for submitting code & software for further information.

Data

Policy information about availability of data

All manuscripts must include a data availability statement. This statement should provide the following information, where applicable:

- Accession codes, unique identifiers, or web links for publicly available datasets
- A description of any restrictions on data availability
- For clinical datasets or third party data, please ensure that the statement adheres to our policy

The cryo-EM map and atomic coordinate of the ARMH2-EFCAB9-CATSPER subcomplex have been deposited at the Electron Microscopy Data Bank (EMDB) and the Protein Data Bank (PDB) under the accession codes of EMD-63452 [https://www.ebi.ac.uk/pdbe/entry/emdb/EMD-63452] and 9LWO [https://doi.org/10.2210/pdb9lwo/pdb], respectively. The proteomics raw data have been deposited in iProX under the accession code of IPX0013365000 [https://www.iprox.cn//page/

SCV017.html?query=IPX0013365000]. The source data underlying Figures 2a,e,f, 4a-e, 5b,d-f, 6a,b,d-f and Supplementary Figures 5b-d, 6a-c, 7b, 8a are provided as a Source Data file.

Research involving human participants, their data, or biological material

Policy information about studies with human participants or human data. See also policy information about sex, gender (identity/presentation), and sexual orientation and race, ethnicity and racism.

Reporting on sex and gender	N/A
Reporting on race, ethnicity, or other socially relevant groupings	N/A
Population characteristics	N/A
Recruitment	N/A
Ethics oversight	N/A

Note that full information on the approval of the study protocol must also be provided in the manuscript.

Field-specific reporting

Please select the one below that is the best fit for your research. If you are not sure, read the appropriate sections before making your selection.

☒ Life sciences ☐ Behavioural & social sciences ☐ Ecological, evolutionary & environmental sciences

For a reference copy of the document with all sections, see nature.com/documents/nr-reporting-summary-flat.pdf

Life sciences study design

All studies must disclose on these points even when the disclosure is negative.

Sample size	For cryo-EM analysis, a total of 34,493 movie stacks were collected in this study. For experiments involving western blotting, histology, mating tests, in vitro fertilization (IVF) and embryo culture in vitro, quantitative proteomic analysis, and super-resolution imaging, a minimum of n = 3 male mice were used as both biological and technical replicates. For computer-assisted sperm analysis (CASA), n = 5 views per mouse and a total of 5–6 animals were selected as replicates. For flagellar bending waveform analysis, n = 15–20 sperm cells per group from 3 animals were analyzed. For electrophysiological recordings, n = 5–10 sperm cells from at least 3 mice were used. Additionally, several million sperm cells per group were utilized for lysate preparation and imaging. These sample sizes were determined to be sufficient based on internal controls and the low variability observed in the data.
Data exclusions	For cryo-EM analysis, only high-resolution and homogeneous particles were retained to ensure the generation of high-quality maps, while micrographs with poor CTF fitting were excluded. For all other datasets, no data points were excluded from the analysis.
Replication	All attempts to replicate the results, including protein purifications and pull-down assays, were successfully conducted following the protocol outlined in the Methods section. The number of replicates performed is detailed in the corresponding figure legends.
Randomization	For animal studies, mice were grouped based on their genotype rather than being randomly assigned to experimental groups. For electrophysiology study, the sperm were randomly selected. For immunocytochemistry, flagellar bending waveform, and sperm motility analyses, data were collected following a randomized approach that avoided any systematic bias regarding sample location on grids/ coverslips, size, or microscopic fields. For immunoblotting, millions of sperm were randomly isolated from the suspension and subsequently lysed for gel electrophoresis.
Blinding	Blinding was not applicable to the mouse experiments, as mice were grouped based on genotype. However, all analyses adhered to pre-established protocols to minimize bias.

Reporting for specific materials, systems and methods

We require information from authors about some types of materials, experimental systems and methods used in many studies. Here, indicate whether each material, system or method listed is relevant to your study. If you are not sure if a list item applies to your research, read the appropriate section before selecting a response.

Materials & experimental systems

n/a	Involved in the study	
	x	Antibodies
	x	Eukaryotic cell lines
x		Palaeontology and archaeology
	x	Animals and other organisms
x		Clinical data
x		Dual use research of concern
x		Plants

Methods

n/a	Involved in the study	
x		ChIP-seq
x		Flow cytometry
x		MRI-based neuroimaging

Antibodies

Antibodies used	For Western bloting:<br>Primary antibodies: Rabbit polyclonal anti-mCATSPER1 and mCATSPERβ are homemade in Wu lab. Mouse anti-mARMH2 is homemade in Liu lab. Rabbit anti-mCATSPERζ, mCATSPERθ, mCATSPERγ, mCATSPERε, mEFCAB9 and mCATSPERδ antibodies are from Chung lab. Other antibodies are from commercial sources: Rabbit anti-GFP antibody (Thermo Fisher, Cat# A-11122), Mouse anti-β actin clone AC-15, purified from hybridoma cell culture (Sigma-aldrich, Cat# A3854-200UL), Mouse anti-Acetylated Tubulin antibody (Sigma-aldrich, Cat# T7451), Rabbit anti-FLAG antibody (HUABIO, Cat# 0912-1), Rabbit anti-Strep antibody (HUABIO, Cat# HA500061). Secondary antibodies: . HRP Conjugated Goat anti-Rabbit IgG h+l antibody (Absin, Cat# abs20040-500uL), Goat Anti-Mouse IgG HRP antibody (Abmart, Cat#M21001L)<br><br>For super-resolution imaging:<br>Rabbit anti-GFP antibody (Thermo Fisher, Cat# A-11122)<br>Goat anti-rabbit IgG (H+L) Alexa Fluor™ 647 (Thermo Fisher, Cat# A21245)
Validation	The specificity of homemade antibodies was confirmed using knockout animals, which showed no signal. Commercial antibodies were validated as per the manufacturers' specifications on their respective websites.

Eukaryotic cell lines

Policy information about cell lines and Sex and Gender in Research

Cell line source(s)	HEK293F cell line was purchased from Thermo Fisher Scientific Inc. (Cat#R79007, RRID: CVCL_D603)
Authentication	Since the proteins used in this study were successfully expressed in HEK293F cells, no further authentication of the commercially available HEK293F cell line was necessary.
Mycoplasma contamination	The cell line was not tested for mycoplasma contamination since no observed contamination.
Commonly misidentified lines<br>(See ICLAC register)	There is no commonly misidentified cell lines used in this study.

Animals and other research organisms

Policy information about studies involving animals; ARRIVE guidelines recommended for reporting animal research, and Sex and Gender in Research

Laboratory animals	The description of research animals used in this study is included in the Methods, section "Animals" and "Generation of knockout mice" (p.18). Wild-type, Catsper1⁻/⁻, Catsperq⁻/⁻, Efcab9 ⁻/⁻, C2cd6⁻/⁻, and Armh2⁻/⁻ male C57BL/6 mice (8-24 weeks old) were utilized for sperm and testis collection. The mice were housed under a 12/12-hour light/dark cycle at 20-26°C, and humidity 40-70% with 2-5 animals per cage.
Wild animals	The study did not involve wild animals.
Reporting on sex	Only male mouse is considered in this study since CatSper is sperm-specific.
Field-collected samples	No field-collected samples were used in this study.
Ethics oversight	The animal maintenance and experimental procedures were performed in accordance with institutional guidelines, and all animal studies were approved by the Institutional Animal Care and Use Committee (IACUC) of Westlake University in Hangzhou, China.

Note that full information on the approval of the study protocol must also be provided in the manuscript.

Plants

Seed stocks	N/A
Novel plant genotypes	N/A
Authentication	N/A
